# 3-Amino­pyridinium *trans*-diaqua­dioxalato­chromate(III)

**DOI:** 10.1107/S1600536812025020

**Published:** 2012-06-13

**Authors:** Ichraf Chérif, Mohamed Faouzi Zid, Malika ­El-Ghozzi, Daniel Avignant

**Affiliations:** a Laboratoire de Matériaux et Cristallochimie, Faculté des Sciences de Tunis, Université de Tunis El Manar, 2092 Manar II Tunis, Tunisia; b Clermont Université, Université Blaise Pascal, Institut de Chimie de Clermont-Ferrand, BP 10448, 63000 Clermont-Ferrand, France; c CNRS, UMR 6296, ICCF, BP 80026, 63171 Aubière, France

## Abstract

In the structure of the title compound, (C_5_H_7_N_2_)[Cr(C_2_O_4_)_2_(H_2_O)_2_], two crystallographically independent formula units are present. Both chromium atoms are six-coordinated in a distorted octa­hedral geometry by two chelating equatorial oxalato ligands and two axial water mol­ecules. The [Cr(C_2_O_4_)_2_(H_2_O)_2_]^−^ anions and C_5_H_7_N_2_^+^ cations are linked through a complex three-dimensional hydrogen-bonding network consisting of N—H⋯O and O—H⋯O inter­actions.

## Related literature

For the versatility of the oxalato ligand, see: Hernández-Molina *et al.* (2001 ▶); Martak *et al.* (2009 ▶); Marinescu *et al.* (2011 ▶). For magnetic studies of oxalatochromium (III) complexes, see: Chen *et al.* (2005 ▶); Marinescu *et al.* (2011 ▶). For complexes containing the [Cr(C_2_O_4_)_2_(H_2_O)_2_]^−^ motif com­pleted by various uncoordinated cations including quinolin­ium, 4-dimethyl­amino­pyridinium and 4-amino­pyridinium, see: Bélombé *et al.* (2009 ▶); Nenwa *et al.* (2010 ▶); Chérif *et al.* (2011 ▶).
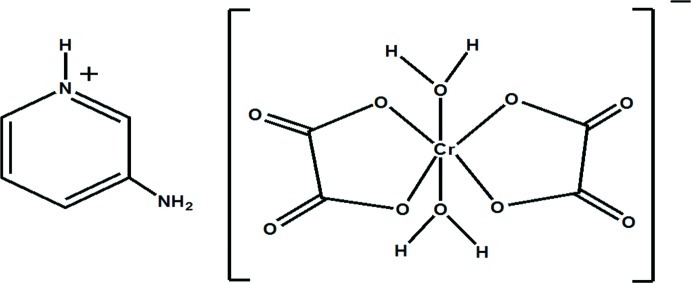


## Experimental

### 

#### Crystal data

(C_5_H_7_N_2_)[Cr(C_2_O_4_)_2_(H_2_O)_2_]
*M* *_r_* = 359.20
Monoclinic, 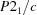

*a* = 7.3901 (15) Å
*b* = 17.586 (4) Å
*c* = 20.995 (5) Å
β = 107.941 (11)°
*V* = 2596.0 (10) Å^3^
*Z* = 8
Mo *K*α radiation
μ = 0.94 mm^−1^
*T* = 296 K
0.53 × 0.33 × 0.19 mm

#### Data collection

Bruker APEXII CCD diffractometer
Absorption correction: multi-scan (*SADABS*; Bruker, 2008 ▶) *T*_min_ = 0.695, *T*_max_ = 0.835
22038 measured reflections
5865 independent reflections
4091 reflections with *I* > 2σ(*I*)
*R*_int_ = 0.036

#### Refinement

*R*[*F* ^2^ > 2σ(*F* ^2^)] = 0.039
*wR*(*F* ^2^) = 0.112
*S* = 1.04
5865 reflections
421 parameters
12 restraints
H atoms treated by a mixture of independent and constrained refinement
Δρ_max_ = 0.68 e Å^−3^
Δρ_min_ = −0.42 e Å^−3^

### 

Data collection: *APEX2* (Bruker, 2008 ▶); cell refinement: *SAINT* (Bruker, 2008 ▶); data reduction: *SAINT*; program(s) used to solve structure: *SHELXS97* (Sheldrick, 2008 ▶); program(s) used to refine structure: *SHELXL97* (Sheldrick, 2008 ▶); molecular graphics: *DIAMOND* (Brandenburg, 1998 ▶); software used to prepare material for publication: *SHELXTL* (Sheldrick, 2008 ▶).

## Supplementary Material

Crystal structure: contains datablock(s) I, global. DOI: 10.1107/S1600536812025020/vn2039sup1.cif

Structure factors: contains datablock(s) I. DOI: 10.1107/S1600536812025020/vn2039Isup2.hkl

Additional supplementary materials:  crystallographic information; 3D view; checkCIF report

## Figures and Tables

**Table 1:** Selected bond lengths (Å)

Cr1—O11	2.0223 (18)
Cr1—O12	2.0017 (17)
Cr1—O13	1.9421 (17)
Cr1—O14	1.9690 (18)
Cr1—O15	1.9771 (18)
Cr1—O16	1.9517 (18)
Cr2—O21	2.006 (2)
Cr2—O22	2.007 (2)
Cr2—O23	1.9604 (17)
Cr2—O24	1.9846 (18)
Cr2—O25	1.9429 (17)
Cr2—O26	1.9793 (18)

**Table 2:** Hydrogen-bond geometry (Å, °)

*D*—H⋯*A*	*D*—H	H⋯*A*	*D*⋯*A*	*D*—H⋯*A*
N11—H11*A*⋯O25^i^	0.86	2.22	3.047 (4)	161
N11—H11*B*⋯O22^ii^	0.86	2.46	3.139 (4)	136
N12—H12⋯O18^iii^	0.86	2.30	2.998 (3)	139
N12—H12⋯O20^iii^	0.86	2.16	2.896 (3)	144
N21—H21*A*⋯O13^iv^	0.86	2.31	3.142 (3)	163
N21—H21*B*⋯O11^v^	0.86	2.42	3.201 (4)	151
N22—H22⋯O28^vi^	0.86	2.38	3.121 (3)	145
N22—H22⋯O30^vi^	0.86	2.05	2.755 (3)	138
O11—H111⋯O27^vii^	0.84 (3)	2.30 (3)	3.103 (3)	161 (2)
O11—H111⋯O29^vii^	0.84 (3)	2.19 (2)	2.749 (3)	123 (2)
O11—H211⋯O18^viii^	0.84 (1)	1.95 (2)	2.777 (3)	173 (3)
O12—H112⋯O27	0.84 (2)	1.85 (2)	2.684 (3)	176 (2)
O12—H212⋯O28^ii^	0.84 (1)	1.78 (1)	2.619 (2)	172 (3)
O21—H121⋯O20^iii^	0.84 (2)	1.85 (1)	2.682 (3)	176 (3)
O21—H221⋯O17^v^	0.84 (2)	1.85 (2)	2.681 (3)	177 (2)
O22—H122⋯O23^ix^	0.85 (2)	1.94 (2)	2.780 (2)	173 (3)
O22—H222⋯O19	0.84 (2)	1.85 (2)	2.646 (3)	157 (3)

Symmetry codes: (i) 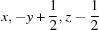; (ii) 
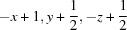
; (iii) 
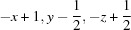
; (iv) 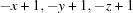; (v) 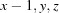; (vi) 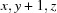; (vii) 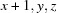; (viii) 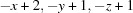; (ix) 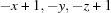.

**Fig. 1.**
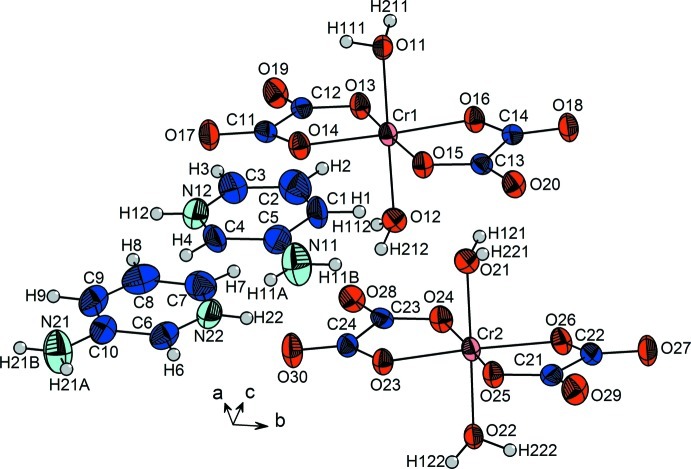
A view of the title compound with the atom-numbering scheme. Thermal ellipsoids are drawn at the 50% probability level for non-H atoms.

**Fig. 2.**
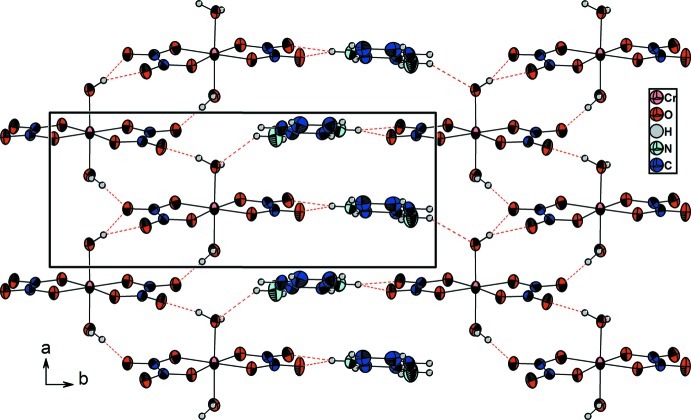
View of the hydrogen bonding system (dashed lines) and π—π stacking interactions between pairs of 3-aminopyridinium cations showing the layered structure.

**Fig. 3.**
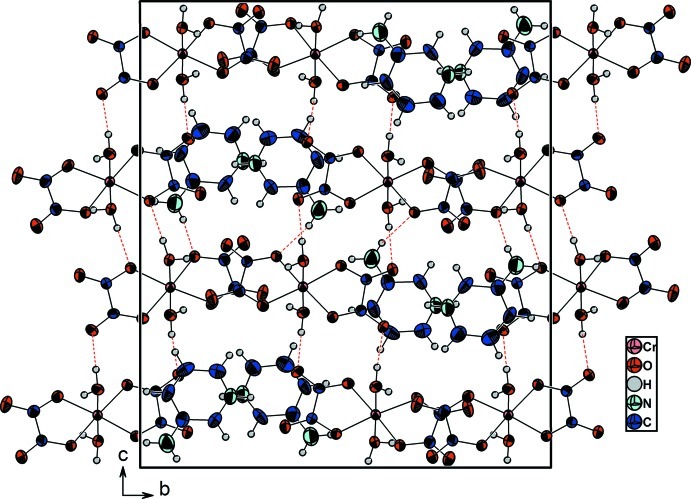
Structure projection along a axis showing connection between layers via hydrogen bonds (dashed lines).

## supplementary crystallographic information

### Comment

In recent years, much work has been focused on the great versatility of the oxalato ligand which affords a wide number of homo and heteropolynuclear complexes with various dimensionalities and network architectures (Marinescu *et al.*, 2011; Martak *et al.*, 2009; Hernández-Molina *et al.*, 2001). This exceptional versatility of the oxalato ligands offers possibilities for the tuning of magnetic properties and thus makes them very useful for obtaining materials with potential applications in magnetism (Chen *et al.*, 2005; Marinescu *et al.*, 2011). The present investigation deals with the new oxalato chromium (III) salt: (C_5_H_7_N_2_)[Cr(C_2_O_4_)_2_(H_2_O)_2_].

The structure is made up of two crystallographically independent entities of formula (C_5_H_7_N_2_)[Cr(C_2_O_4_)_2_(H_2_O)_2_] (Fig. 1). The two chromium atoms Cr1 and Cr2 exhibit a distorted octahedral environment with bond lengths and angles very close to each other. The Cr—O(ox) bonds ranges from 1.9421 (17) to 1.9771 (18) Å for Cr1—O(ox) and from 1.9429 (17) to 1.9846 (18) Å for Cr2—O(ox), values which are shorter than the Cr—O(water) bonds [2.0017 (17) - 2.0223 (18) Å for Cr1—O(water) and 2.006 (2) - 2.007 (2) Å for Cr2—O(water)]. Such type of coordination was already observed in complexes containing the [Cr(C_2_O_4_)_2_(H_2_O)_2_]^-^ motif completed with various uncoordinated cations including quinolinium, 4-dimethylaminopyridinium and 4-aminopyridinium with similar geometric parameters (Bélombé *et al.*, 2009; Nenwa *et al.*, 2010; Chérif *et al.*, 2011). The charge balance of the mononuclear anion is provided by an uncoordinated 3-aminopyridinium cation.

In the crystal structure intermolecular hydrogen bonds and very weak π—π contacts established between the 3-aminopyridinium cations connect the ionic entities generating layers parallel to (001) (Fig. 2). These layers are further connected by O—H···O [O11—H211···O18 / O12—H212···O28 / O21—H121···O20 / O22—H122···O23] and N—H···O [N11—H11A···O25 / N21—H21···O13] hydrogen bonds (Fig. 3). The shortest interlayer chromium (III) ion separations are: Cr1—Cr1: 5.808 (2) Å, Cr2—Cr2: 5.226 (2) Å and Cr1—Cr2: 6.301 (2) Å.

### Experimental

To 10 cm^3^ of a solution of chromium chloride CrCl_3_.6H_2_O (1 mmol) was added under stirring at room temperature previously prepared solutions of 3-aminopyridine C_5_H_6_N_2_ (1 mmol, 10 cm^3^) and oxalic acid H_2_C_2_O_4_.2H_2_O (2 mmol, 10 cm^3^) in water. The obtained solution was stirred for 3 h at 323 K and prismatic violet single crystals were grown by slow evaporation at room temperature.

### Refinement

All non hydrogen atoms were refined anisotropically. The hydrogen atoms of the water molecules were located in a difference Fourier map and refined with restraints: d(O—H) = 0.85 (1) Å, d(H···H) = 1.387 (2) Å and *U*_iso_(H) = 1.5*U*_eq_(O), whereas those of the 3-aminopyridinium cation were set in calculated positions and refined as riding atoms with d(C—H) = 0.93 Å, d(N—H) = 0.86 Å and *U*_iso_(H) = 1.2*U*_eq_(C or N). The highest residual peak in the final Fourier map was located at 0.97 Å from the C4 atom and the deepest hole was located at 0.62 Å from the Cr2 atom.

### Crystal data

**Table:**

(C_5_H_7_N_2_)[Cr(C_2_O_4_)_2_(H_2_O)_2_]	*F*(000) = 1464
*M**_r_* = 359.20	*D*_x_ = 1.838 Mg m^−^^3^
Monoclinic, *P*2_1_/*c*	Mo *K*α radiation, λ = 0.71073 Å
Hall symbol: -P 2ybc	Cell parameters from 5082 reflections
*a* = 7.3901 (15) Å	θ = 3.0–27.3°
*b* = 17.586 (4) Å	µ = 0.94 mm^−^^1^
*c* = 20.995 (5) Å	*T* = 296 K
β = 107.941 (11)°	Prism, violet
*V* = 2596.0 (10) Å^3^	0.53 × 0.33 × 0.19 mm
*Z* = 8	

### Data collection

**Table:**

Bruker APEXII CCD diffractometer	5865 independent reflections
Radiation source: fine-focus sealed tube	4091 reflections with *I* > 2σ(*I*)
Graphite monochromator	*R*_int_ = 0.036
φ and ω scans	θ_max_ = 27.5°, θ_min_ = 1.5°
Absorption correction: multi-scan (*SADABS*; Bruker, 2008)	*h* = −9→9
*T*_min_ = 0.695, *T*_max_ = 0.835	*k* = −22→22
22038 measured reflections	*l* = −24→27

### Refinement

**Table:**

Refinement on *F*^2^	Primary atom site location: structure-invariant direct methods
Least-squares matrix: full	Secondary atom site location: difference Fourier map
*R*[*F*^2^ > 2σ(*F*^2^)] = 0.039	Hydrogen site location: inferred from neighbouring sites
*wR*(*F*^2^) = 0.112	H atoms treated by a mixture of independent and constrained refinement
*S* = 1.04	*w* = 1/[σ^2^(*F*_o_^2^) + (0.0522*P*)^2^ + 1.1562*P*] where *P* = (*F*_o_^2^ + 2*F*_c_^2^)/3
5865 reflections	(Δ/σ)_max_ = 0.001
421 parameters	Δρ_max_ = 0.68 e Å^−^^3^
12 restraints	Δρ_min_ = −0.42 e Å^−^^3^

### Special details

**Table:**

Geometry. Bond distances, angles etc. have been calculated using the rounded fractional coordinates. All su's are estimated from the variances of the (full) variance-covariance matrix. The cell esds are taken into account in the estimation of distances, angles and torsion angles
Refinement. Refinement of *F*^2^ against ALL reflections. The weighted *R*-factor *wR* and goodness of fit *S* are based on *F*^2^, conventional *R*-factors *R* are based on *F*, with *F* set to zero for negative *F*^2^. The threshold expression of *F*^2^ > σ(*F*^2^) is used only for calculating *R*-factors(gt) *etc*. and is not relevant to the choice of reflections for refinement. *R*-factors based on *F*^2^ are statistically about twice as large as those based on *F*, and *R*- factors based on ALL data will be even larger.

### Fractional atomic coordinates and isotropic or equivalent isotropic displacement parameters (Å^2^)

**Table:**

	*x*	*y*	*z*	*U*_iso_*/*U*_eq_	
Cr1	0.86991 (5)	0.39632 (2)	0.387432 (18)	0.02059 (12)	
O11	1.1473 (2)	0.39735 (10)	0.44297 (9)	0.0262 (4)	
H111	1.205 (4)	0.3619 (12)	0.4303 (12)	0.039*	
H211	1.160 (4)	0.3939 (15)	0.4838 (6)	0.039*	
O12	0.5955 (2)	0.39686 (10)	0.33227 (8)	0.0265 (4)	
H112	0.531 (3)	0.3644 (13)	0.3449 (12)	0.040*	
H212	0.575 (4)	0.3931 (16)	0.2906 (5)	0.040*	
O13	0.8080 (2)	0.33173 (10)	0.45283 (8)	0.0252 (4)	
O14	0.9080 (2)	0.29864 (10)	0.34754 (8)	0.0276 (4)	
O15	0.9201 (2)	0.46853 (10)	0.32268 (8)	0.0255 (4)	
O16	0.8323 (2)	0.48926 (10)	0.43257 (8)	0.0252 (4)	
O17	0.8865 (3)	0.17386 (11)	0.36410 (10)	0.0379 (5)	
O18	0.8431 (3)	0.61488 (10)	0.42415 (9)	0.0320 (4)	
O19	0.7775 (3)	0.21093 (10)	0.47675 (9)	0.0359 (5)	
O20	0.9095 (3)	0.59268 (10)	0.30172 (9)	0.0304 (4)	
C11	0.8736 (3)	0.24077 (15)	0.37920 (12)	0.0252 (6)	
C12	0.8138 (3)	0.26020 (14)	0.44180 (12)	0.0228 (5)	
C13	0.8984 (3)	0.53796 (14)	0.33634 (12)	0.0223 (5)	
C14	0.8543 (3)	0.55075 (14)	0.40331 (12)	0.0228 (5)	
Cr2	0.37874 (5)	0.07363 (2)	0.386675 (19)	0.02155 (12)	
O21	0.1084 (3)	0.07383 (10)	0.32683 (9)	0.0272 (4)	
H121	0.103 (4)	0.0772 (16)	0.2865 (6)	0.041*	
H221	0.036 (3)	0.1047 (13)	0.3372 (12)	0.041*	
O22	0.6505 (3)	0.07089 (10)	0.44535 (8)	0.0259 (4)	
H122	0.663 (4)	0.0556 (14)	0.4848 (7)	0.039*	
H222	0.704 (4)	0.1132 (9)	0.4470 (13)	0.039*	
O23	0.3433 (2)	−0.02304 (9)	0.42779 (8)	0.0258 (4)	
O24	0.4379 (2)	0.00638 (10)	0.31981 (8)	0.0270 (4)	
O25	0.3026 (2)	0.13247 (10)	0.45248 (8)	0.0263 (4)	
O26	0.4174 (2)	0.17523 (10)	0.35243 (9)	0.0288 (4)	
O27	0.3779 (3)	0.29802 (11)	0.37330 (10)	0.0385 (5)	
O28	0.4338 (3)	−0.11727 (10)	0.29469 (8)	0.0315 (4)	
O29	0.2676 (3)	0.25175 (11)	0.48140 (10)	0.0375 (5)	
O30	0.3577 (3)	−0.14718 (10)	0.41233 (9)	0.0404 (5)	
C21	0.3098 (3)	0.20509 (15)	0.44561 (13)	0.0252 (6)	
C22	0.3738 (3)	0.22954 (15)	0.38542 (13)	0.0267 (6)	
C23	0.4178 (3)	−0.06459 (15)	0.33022 (11)	0.0227 (5)	
C24	0.3688 (3)	−0.08241 (14)	0.39500 (12)	0.0246 (6)	
N11	0.1544 (4)	0.41533 (17)	0.06676 (15)	0.0610 (8)	
H11A	0.1725	0.3951	0.0319	0.073*	
H11B	0.1520	0.4640	0.0704	0.073*	
N12	0.1098 (3)	0.24902 (15)	0.15746 (14)	0.0446 (6)	
H12	0.1131	0.2006	0.1524	0.053*	
C1	0.1011 (4)	0.39996 (18)	0.17377 (15)	0.0418 (8)	
H1	0.0967	0.4522	0.1799	0.050*	
C2	0.0795 (5)	0.3528 (2)	0.22143 (17)	0.0523 (9)	
H2	0.0636	0.3732	0.2602	0.063*	
C3	0.0805 (4)	0.27507 (19)	0.21400 (16)	0.0485 (8)	
H3	0.0619	0.2423	0.2462	0.058*	
C4	0.1338 (4)	0.29243 (18)	0.10945 (16)	0.0402 (8)	
H4	0.1532	0.2702	0.0719	0.048*	
C5	0.1303 (4)	0.37163 (18)	0.11460 (15)	0.0396 (7)	
N21	0.3221 (4)	0.56454 (17)	0.44750 (14)	0.0603 (8)	
H21A	0.3077	0.5887	0.4812	0.072*	
H21B	0.3170	0.5157	0.4464	0.072*	
N22	0.3907 (3)	0.71990 (14)	0.34738 (13)	0.0405 (6)	
H22	0.3952	0.7687	0.3499	0.049*	
C6	0.3605 (4)	0.68177 (17)	0.39823 (15)	0.0359 (7)	
H6	0.3450	0.7080	0.4346	0.043*	
C7	0.4141 (4)	0.68807 (19)	0.29359 (16)	0.0454 (8)	
H7	0.4340	0.7173	0.2595	0.055*	
C8	0.4078 (4)	0.6097 (2)	0.28982 (15)	0.0464 (8)	
H8	0.4236	0.5853	0.2526	0.056*	
C9	0.3789 (4)	0.56839 (17)	0.34007 (16)	0.0412 (7)	
H9	0.3767	0.5156	0.3370	0.049*	
C10	0.3523 (4)	0.60299 (17)	0.39666 (15)	0.0350 (7)	

### Atomic displacement parameters (Å^2^)

**Table:**

	*U*^11^	*U*^22^	*U*^33^	*U*^12^	*U*^13^	*U*^23^
Cr1	0.0306 (2)	0.0155 (2)	0.0179 (2)	−0.00071 (16)	0.01067 (16)	0.00029 (15)
O11	0.0333 (10)	0.0199 (10)	0.0251 (9)	0.0027 (8)	0.0085 (8)	−0.0015 (7)
O12	0.0326 (10)	0.0269 (10)	0.0212 (9)	−0.0032 (8)	0.0100 (8)	0.0025 (8)
O13	0.0388 (10)	0.0196 (9)	0.0211 (9)	−0.0010 (8)	0.0149 (8)	−0.0010 (7)
O14	0.0389 (10)	0.0231 (10)	0.0253 (9)	−0.0014 (8)	0.0163 (8)	−0.0023 (8)
O15	0.0373 (10)	0.0200 (10)	0.0227 (9)	0.0000 (8)	0.0144 (8)	0.0008 (7)
O16	0.0383 (10)	0.0201 (9)	0.0206 (9)	0.0001 (8)	0.0142 (8)	−0.0006 (7)
O17	0.0400 (11)	0.0233 (11)	0.0496 (12)	0.0021 (9)	0.0129 (9)	−0.0104 (9)
O18	0.0489 (11)	0.0189 (10)	0.0288 (10)	0.0010 (8)	0.0128 (9)	−0.0030 (8)
O19	0.0536 (12)	0.0226 (10)	0.0324 (11)	−0.0083 (9)	0.0148 (9)	0.0037 (8)
O20	0.0459 (11)	0.0210 (10)	0.0247 (10)	−0.0048 (8)	0.0116 (8)	0.0041 (8)
C11	0.0212 (13)	0.0257 (15)	0.0268 (13)	−0.0005 (11)	0.0043 (10)	−0.0032 (11)
C12	0.0237 (12)	0.0203 (14)	0.0224 (12)	−0.0013 (10)	0.0041 (10)	0.0004 (10)
C13	0.0232 (12)	0.0211 (13)	0.0210 (12)	−0.0027 (10)	0.0046 (10)	0.0002 (10)
C14	0.0247 (13)	0.0206 (14)	0.0204 (12)	0.0005 (10)	0.0030 (10)	0.0001 (10)
Cr2	0.0317 (2)	0.0164 (2)	0.0186 (2)	0.00061 (16)	0.01077 (17)	0.00100 (15)
O21	0.0335 (10)	0.0263 (10)	0.0230 (9)	0.0036 (8)	0.0102 (8)	−0.0026 (8)
O22	0.0352 (10)	0.0178 (9)	0.0247 (9)	−0.0015 (8)	0.0092 (8)	0.0025 (7)
O23	0.0392 (10)	0.0200 (10)	0.0212 (9)	−0.0015 (8)	0.0139 (8)	−0.0004 (7)
O24	0.0387 (10)	0.0258 (10)	0.0208 (9)	0.0008 (8)	0.0157 (8)	0.0000 (7)
O25	0.0385 (10)	0.0197 (9)	0.0233 (9)	0.0011 (8)	0.0133 (8)	0.0005 (7)
O26	0.0370 (10)	0.0247 (10)	0.0273 (10)	0.0011 (8)	0.0138 (8)	0.0069 (8)
O27	0.0391 (11)	0.0206 (10)	0.0539 (13)	−0.0015 (9)	0.0114 (10)	0.0111 (9)
O28	0.0457 (11)	0.0267 (11)	0.0231 (9)	0.0063 (8)	0.0119 (8)	−0.0056 (8)
O29	0.0495 (12)	0.0260 (11)	0.0393 (11)	0.0064 (9)	0.0168 (9)	−0.0068 (9)
O30	0.0722 (14)	0.0195 (11)	0.0342 (11)	−0.0016 (10)	0.0234 (10)	0.0006 (8)
C21	0.0230 (13)	0.0220 (14)	0.0275 (13)	0.0017 (11)	0.0032 (10)	−0.0010 (11)
C22	0.0233 (13)	0.0239 (14)	0.0298 (14)	0.0007 (11)	0.0037 (11)	0.0053 (11)
C23	0.0256 (13)	0.0244 (14)	0.0168 (12)	0.0034 (10)	0.0045 (10)	0.0009 (10)
C24	0.0312 (14)	0.0198 (14)	0.0224 (13)	−0.0002 (11)	0.0077 (11)	−0.0013 (10)
N11	0.091 (2)	0.0408 (17)	0.063 (2)	−0.0057 (16)	0.0408 (18)	−0.0036 (15)
N12	0.0458 (15)	0.0305 (14)	0.0538 (17)	0.0036 (12)	0.0100 (13)	−0.0005 (13)
C1	0.0465 (18)	0.0302 (16)	0.0490 (19)	0.0060 (14)	0.0154 (15)	−0.0161 (14)
C2	0.057 (2)	0.058 (2)	0.047 (2)	0.0019 (18)	0.0239 (16)	−0.0090 (17)
C3	0.0514 (19)	0.045 (2)	0.047 (2)	0.0028 (16)	0.0122 (16)	−0.0084 (16)
C4	0.0322 (15)	0.0386 (19)	0.0459 (18)	0.0040 (13)	0.0063 (13)	−0.0226 (15)
C5	0.0374 (16)	0.0363 (18)	0.0443 (18)	0.0008 (13)	0.0113 (14)	0.0005 (14)
N21	0.087 (2)	0.0436 (18)	0.065 (2)	−0.0002 (15)	0.0444 (17)	0.0071 (15)
N22	0.0423 (14)	0.0288 (14)	0.0484 (16)	−0.0035 (11)	0.0108 (12)	−0.0017 (12)
C6	0.0349 (15)	0.0326 (17)	0.0402 (17)	0.0000 (13)	0.0114 (13)	−0.0127 (13)
C7	0.0472 (18)	0.051 (2)	0.0379 (18)	−0.0001 (16)	0.0129 (15)	0.0033 (15)
C8	0.0449 (18)	0.060 (2)	0.0375 (18)	0.0007 (16)	0.0169 (14)	−0.0205 (16)
C9	0.0388 (17)	0.0286 (16)	0.056 (2)	0.0046 (13)	0.0140 (15)	−0.0146 (15)
C10	0.0307 (15)	0.0317 (16)	0.0446 (18)	0.0006 (12)	0.0143 (13)	−0.0036 (13)

### Geometric parameters (Å, º)

**Table:**

Cr1—O11	2.0223 (18)	O22—H122	0.848 (16)
Cr1—O12	2.0017 (17)	O22—H222	0.84 (2)
Cr1—O13	1.9421 (17)	N11—C5	1.320 (4)
Cr1—O14	1.9690 (18)	N12—C3	1.351 (4)
Cr1—O15	1.9771 (18)	N12—C4	1.319 (4)
Cr1—O16	1.9517 (18)	N11—H11B	0.8600
Cr2—O21	2.006 (2)	N11—H11A	0.8600
Cr2—O22	2.007 (2)	N12—H12	0.8600
Cr2—O23	1.9604 (17)	N21—C10	1.339 (4)
Cr2—O24	1.9846 (18)	N22—C6	1.336 (4)
Cr2—O25	1.9429 (17)	N22—C7	1.319 (4)
Cr2—O26	1.9793 (18)	N21—H21A	0.8600
O13—C12	1.282 (3)	N21—H21B	0.8600
O14—C11	1.283 (3)	N22—H22	0.8600
O15—C13	1.276 (3)	C11—C12	1.548 (3)
O16—C14	1.279 (3)	C13—C14	1.554 (3)
O17—C11	1.230 (3)	C21—C22	1.541 (4)
O18—C14	1.222 (3)	C23—C24	1.544 (3)
O19—C12	1.218 (3)	C1—C2	1.347 (5)
O20—C13	1.224 (3)	C1—C5	1.415 (4)
O11—H211	0.835 (13)	C2—C3	1.376 (5)
O11—H111	0.84 (3)	C4—C5	1.398 (4)
O12—H212	0.843 (11)	C1—H1	0.9300
O12—H112	0.84 (2)	C2—H2	0.9300
O23—C24	1.296 (3)	C3—H3	0.9300
O24—C23	1.284 (3)	C4—H4	0.9300
O25—C21	1.288 (3)	C6—C10	1.387 (4)
O26—C22	1.278 (3)	C7—C8	1.380 (5)
O27—C22	1.233 (3)	C8—C9	1.351 (4)
O28—C23	1.218 (3)	C9—C10	1.402 (4)
O29—C21	1.216 (3)	C6—H6	0.9300
O30—C24	1.206 (3)	C7—H7	0.9300
O21—H121	0.837 (15)	C8—H8	0.9300
O21—H221	0.84 (2)	C9—H9	0.9300
			
Cr1···O28^i^	3.7984 (19)	O19···H122	2.88 (3)
Cr2···O19	3.829 (2)	O19···H6^ii^	2.7100
Cr1···H21A^ii^	3.4100	O19···H222	1.852 (18)
Cr1···H2^iii^	3.4200	O19···H4^vi^	2.9000
O11···O14	2.824 (3)	O20···H12^i^	2.1600
O11···O15	2.855 (2)	O20···H121^i^	1.846 (15)
O11···O13	2.825 (2)	O20···H3^i^	2.8500
O11···O27^iii^	3.103 (3)	O21···H1^xi^	2.6000
O11···O29^iii^	2.749 (3)	O22···H11B^v^	2.4600
O11···N21^iii^	3.201 (4)	O23···H122	2.68 (3)
O11···C14	3.398 (3)	O23···H122^vii^	1.937 (17)
O11···O16	2.787 (2)	O24···H8^v^	2.5000
O11···O18^iv^	2.777 (3)	O24···H121	2.66 (3)
O11···C14^iv^	3.358 (3)	O25···H11A^ix^	2.2200
O12···O27	2.684 (3)	O25···H122	2.88 (3)
O12···O14	2.822 (2)	O25···H221	2.65 (2)
O12···O15	2.772 (2)	O26···H121	2.88 (3)
O12···O13	2.781 (2)	O26···H222	2.65 (3)
O12···O28^i^	2.619 (2)	O27···H112	1.85 (2)
O12···C11	3.389 (3)	O27···H111^viii^	2.30 (3)
O12···C12	3.374 (3)	O28···H212^v^	1.781 (13)
O12···C13	3.325 (3)	O28···H22^x^	2.3800
O12···C14	3.384 (3)	O29···H211^viii^	2.63 (3)
O12···O16	2.805 (2)	O29···H4^ix^	2.3400
O13···C11	2.376 (3)	O29···H111^viii^	2.19 (2)
O13···O12	2.781 (2)	O30···H22^x^	2.0500
O13···O18^iv^	3.178 (3)	O30···H6^x^	2.6000
O13···O14	2.603 (2)	O30···H122^vii^	2.74 (2)
O13···O16	2.817 (3)	N11···O22^i^	3.139 (4)
O13···O27	3.158 (3)	N11···C23^i^	3.254 (4)
O13···N21^ii^	3.142 (3)	N11···C24^i^	3.364 (4)
O13···O11	2.825 (2)	N11···O25^xii^	3.047 (4)
O14···O13	2.603 (2)	N12···O20^v^	2.896 (3)
O14···C12	2.389 (3)	N12···O18^v^	2.998 (3)
O14···O12	2.822 (2)	N21···O11^viii^	3.201 (4)
O14···O15	3.039 (3)	N21···C13^viii^	3.312 (4)
O14···O11	2.824 (3)	N21···C14^viii^	3.301 (4)
O14···C2^iii^	3.405 (4)	N21···O13^ii^	3.142 (3)
O15···C2^iii^	3.405 (4)	N21···O16^ii^	3.211 (3)
O15···O12	2.772 (2)	N22···O30^xiii^	2.755 (3)
O15···O11	2.855 (2)	N22···O28^xiii^	3.121 (3)
O15···C14	2.388 (3)	C1···O28^i^	3.308 (4)
O15···O16	2.608 (2)	C1···O30^xiv^	3.417 (4)
O15···O14	3.039 (3)	C1···C24^xiv^	3.339 (4)
O16···C13	2.380 (3)	C2···O27	3.410 (4)
O16···O11	2.787 (2)	C2···O15^viii^	3.405 (4)
O16···N21^ii^	3.211 (3)	C2···O14^viii^	3.405 (4)
O16···O12	2.805 (2)	C3···O27	3.413 (4)
O16···O16^iv^	3.159 (2)	C3···O20^v^	3.228 (4)
O16···O15	2.608 (2)	C4···O18^v^	3.217 (4)
O16···O13	2.817 (3)	C4···O19^xv^	3.191 (4)
O17···O25^iii^	3.149 (3)	C4···O29^xii^	3.232 (4)
O17···O21^iii^	2.681 (3)	C5···O28^i^	3.204 (4)
O17···O19	2.800 (3)	C5···C23^i^	3.372 (4)
O17···C21^iii^	3.117 (3)	C6···O30^xiii^	3.023 (4)
O17···C7^v^	3.380 (4)	C6···O29^ii^	3.321 (4)
O18···O13^iv^	3.178 (3)	C7···O17^i^	3.380 (4)
O18···O11^iv^	2.777 (3)	C8···O24^i^	3.394 (4)
O18···O20	2.789 (3)	C8···C14	3.577 (4)
O18···C4^i^	3.217 (4)	C9···C14	3.366 (4)
O18···N12^i^	2.998 (3)	C9···C13^viii^	3.568 (4)
O19···O26	3.164 (3)	C9···O20^viii^	3.338 (4)
O19···O22	2.646 (3)	C10···O20^viii^	3.270 (4)
O19···C22	3.027 (3)	C10···C13^viii^	3.398 (4)
O19···C21	3.315 (3)	C11···O29^iii^	3.045 (3)
O19···C4^vi^	3.191 (4)	C11···C21^iii^	3.156 (3)
O19···O17	2.800 (3)	C12···C22	3.145 (3)
O19···O30^vii^	3.019 (3)	C12···O26	3.309 (3)
O19···Cr2	3.829 (2)	C12···O29^iii^	3.200 (3)
O20···N12^i^	2.896 (3)	C12···O27	3.168 (3)
O20···O21^i^	2.682 (3)	C13···N21^iii^	3.312 (4)
O20···C3^i^	3.228 (4)	C13···C10^iii^	3.398 (4)
O20···O18	2.789 (3)	C13···C9^iii^	3.568 (4)
O20···C10^iii^	3.270 (4)	C14···O11^iv^	3.358 (3)
O20···C9^iii^	3.338 (4)	C14···C9	3.366 (4)
O21···C24	3.407 (3)	C14···C8	3.577 (4)
O21···O20^v^	2.682 (3)	C14···N21^iii^	3.301 (4)
O21···C23	3.325 (3)	C21···C11^viii^	3.156 (3)
O21···O17^viii^	2.681 (3)	C21···O30^vii^	3.385 (3)
O21···O25	2.784 (3)	C21···O19	3.315 (3)
O21···O24	2.753 (3)	C21···O17^viii^	3.117 (3)
O21···O23	2.853 (3)	C22···C12	3.145 (3)
O21···C21	3.388 (3)	C22···O19	3.027 (3)
O21···O26	2.816 (3)	C23···C5^v^	3.372 (4)
O21···C22	3.372 (3)	C23···N11^v^	3.254 (4)
O22···O23	2.739 (3)	C24···C1^xi^	3.339 (4)
O22···C24	3.371 (3)	C24···N11^v^	3.364 (4)
O22···O26	2.841 (3)	C24···O22^vii^	3.405 (3)
O22···C24^vii^	3.405 (3)	C11···H221^iii^	2.93 (2)
O22···N11^v^	3.139 (4)	C12···H112	3.04 (2)
O22···O19	2.646 (3)	C12···H222	2.722 (18)
O22···O25	2.836 (3)	C13···H121^i^	2.667 (16)
O22···O23^vii^	2.780 (2)	C13···H12^i^	2.8700
O22···O24	2.855 (2)	C14···H12^i^	2.9200
O23···O24	2.622 (2)	C14···H211^iv^	2.594 (17)
O23···O22^vii^	2.780 (2)	C21···H221	3.09 (2)
O23···O22	2.739 (3)	C21···H11A^ix^	2.9200
O23···C23	2.393 (3)	C21···H111^viii^	2.86 (2)
O23···O25	2.817 (2)	C22···H112	2.88 (2)
O23···O21	2.853 (3)	C22···H111^viii^	2.93 (2)
O24···O21	2.753 (3)	C23···H22^x^	2.9700
O24···O26	3.062 (3)	C23···H212^v^	2.660 (15)
O24···C24	2.385 (3)	C24···H122^vii^	2.648 (18)
O24···C8^v^	3.394 (4)	C24···H22^x^	2.8100
O24···O23	2.622 (2)	H1···H11B	2.4600
O24···O22	2.855 (2)	H1···O21^xiv^	2.6000
O25···N11^ix^	3.047 (4)	H2···O15^viii^	2.5500
O25···O21	2.784 (3)	H2···O14^viii^	2.7800
O25···O30^vii^	3.168 (3)	H2···Cr1^viii^	3.4200
O25···O23	2.817 (2)	H3···O20^v^	2.8500
O25···O22	2.836 (3)	H3···O14^viii^	2.8800
O25···O26	2.607 (2)	H4···O18^v^	2.7300
O25···O17^viii^	3.149 (3)	H4···H11A	2.3700
O25···C22	2.373 (3)	H4···O29^xii^	2.3400
O26···O22	2.841 (3)	H4···O19^xv^	2.9000
O26···O25	2.607 (2)	H6···H21A	2.3700
O26···O24	3.062 (3)	H6···O30^xiii^	2.6000
O26···C21	2.386 (3)	H6···O19^ii^	2.7100
O26···O19	3.164 (3)	H8···O24^i^	2.5000
O26···O21	2.816 (3)	H9···H21B	2.4700
O26···C12	3.309 (3)	H9···O12	2.6600
O27···C3	3.413 (4)	H11A···H4	2.3700
O27···C12	3.168 (3)	H11A···O25^xii^	2.2200
O27···O13	3.158 (3)	H11A···C21^xii^	2.9200
O27···O29	2.760 (3)	H11B···O22^i^	2.4600
O27···O12	2.684 (3)	H11B···H1	2.4600
O27···C2	3.410 (4)	H12···C13^v^	2.8700
O27···O11^viii^	3.103 (3)	H12···C14^v^	2.9200
O28···O30	2.748 (3)	H12···O18^v^	2.3000
O28···C5^v^	3.204 (4)	H12···O20^v^	2.1600
O28···O12^v^	2.619 (2)	H21A···Cr1^ii^	3.4100
O28···N22^x^	3.121 (3)	H21A···O16^ii^	2.7200
O28···Cr1^v^	3.7984 (19)	H21A···O13^ii^	2.3100
O28···C1^v^	3.308 (4)	H21A···H6	2.3700
O29···C6^ii^	3.321 (4)	H21B···H9	2.4700
O29···C12^viii^	3.200 (3)	H21B···O11^viii^	2.4200
O29···C11^viii^	3.045 (3)	H22···O30^xiii^	2.0500
O29···C4^ix^	3.232 (4)	H22···C23^xiii^	2.9700
O29···O27	2.760 (3)	H22···O28^xiii^	2.3800
O29···O11^viii^	2.749 (3)	H22···C24^xiii^	2.8100
O30···O19^vii^	3.019 (3)	H111···C21^iii^	2.86 (2)
O30···O28	2.748 (3)	H111···C22^iii^	2.93 (2)
O30···C1^xi^	3.417 (4)	H111···O29^iii^	2.19 (2)
O30···N22^x^	2.755 (3)	H111···O27^iii^	2.30 (3)
O30···C21^vii^	3.385 (3)	H112···O27	1.85 (2)
O30···O25^vii^	3.168 (3)	H112···C12	3.04 (2)
O30···C6^x^	3.023 (4)	H112···C22	2.88 (2)
O11···H21B^iii^	2.4200	H121···O20^v^	1.846 (14)
O12···H9	2.6600	H121···C13^v^	2.667 (16)
O13···H21A^ii^	2.3100	H122···O30^vii^	2.74 (2)
O13···H211	2.71 (3)	H122···C24^vii^	2.648 (18)
O13···H112	2.61 (2)	H122···O19	2.88 (3)
O14···H212	2.90 (3)	H122···O23^vii^	1.937 (18)
O14···H111	2.59 (3)	H211···C14^iv^	2.594 (17)
O14···H3^iii^	2.8800	H211···O29^iii^	2.63 (3)
O14···H2^iii^	2.7800	H211···O18^iv^	1.946 (15)
O15···H212	2.77 (3)	H211···O16^iv^	2.69 (2)
O15···H2^iii^	2.5500	H212···O28^i^	1.781 (13)
O16···H211	2.87 (3)	H212···C23^i^	2.660 (15)
O16···H211^iv^	2.69 (2)	H221···C11^viii^	2.93 (2)
O16···H21A^ii^	2.7200	H221···C21	3.09 (2)
O17···H221^iii^	1.85 (2)	H221···O17^viii^	1.85 (2)
O17···H222	2.73 (3)	H222···C12	2.722 (18)
O18···H12^i^	2.3000	H222···O17	2.73 (3)
O18···H211^iv^	1.946 (15)	H222···O19	1.852 (18)
O18···H4^i^	2.7300		
			
O11—Cr1—O12	179.21 (7)	H21A—N21—H21B	120.00
O11—Cr1—O13	90.88 (7)	C10—N21—H21A	120.00
O11—Cr1—O14	90.04 (7)	C7—N22—H22	118.00
O11—Cr1—O15	91.10 (7)	C6—N22—H22	118.00
O11—Cr1—O16	89.04 (7)	O17—C11—C12	119.7 (2)
O12—Cr1—O13	89.69 (7)	O14—C11—O17	125.6 (2)
O12—Cr1—O14	90.58 (7)	O14—C11—C12	114.8 (2)
O12—Cr1—O15	88.30 (7)	O13—C12—C11	113.8 (2)
O12—Cr1—O16	90.37 (7)	O19—C12—C11	121.9 (2)
O13—Cr1—O14	83.46 (7)	O13—C12—O19	124.3 (2)
O13—Cr1—O15	175.36 (7)	O15—C13—C14	114.7 (2)
O13—Cr1—O16	92.68 (7)	O15—C13—O20	125.7 (2)
O14—Cr1—O15	100.73 (7)	O20—C13—C14	119.6 (2)
O14—Cr1—O16	176.02 (7)	O16—C14—C13	113.9 (2)
O15—Cr1—O16	83.16 (7)	O16—C14—O18	125.2 (2)
O23—Cr2—O24	83.29 (7)	O18—C14—C13	120.9 (2)
O23—Cr2—O25	92.38 (7)	O25—C21—C22	113.7 (2)
O23—Cr2—O26	175.27 (7)	O25—C21—O29	124.9 (2)
O24—Cr2—O25	174.57 (7)	O29—C21—C22	121.4 (2)
O24—Cr2—O26	101.13 (7)	O27—C22—C21	118.4 (2)
O25—Cr2—O26	83.30 (7)	O26—C22—O27	126.3 (2)
O22—Cr2—O24	91.32 (7)	O26—C22—C21	115.4 (2)
O21—Cr2—O22	178.47 (8)	O28—C23—C24	118.6 (2)
O21—Cr2—O23	92.00 (7)	O24—C23—C24	114.7 (2)
O21—Cr2—O24	87.24 (7)	O24—C23—O28	126.7 (2)
O21—Cr2—O25	89.64 (7)	O23—C24—C23	114.6 (2)
O21—Cr2—O26	89.90 (7)	O23—C24—O30	124.5 (2)
O22—Cr2—O23	87.31 (7)	O30—C24—C23	120.9 (2)
O22—Cr2—O25	91.75 (7)	C2—C1—C5	121.4 (3)
O22—Cr2—O26	90.89 (7)	C1—C2—C3	121.4 (3)
Cr1—O13—C12	114.70 (15)	N12—C3—C2	116.4 (3)
Cr1—O14—C11	113.24 (15)	N12—C4—C5	120.5 (3)
Cr1—O15—C13	113.39 (15)	C1—C5—C4	115.5 (3)
Cr1—O16—C14	114.63 (15)	N11—C5—C1	123.8 (3)
Cr1—O11—H211	111 (2)	N11—C5—C4	120.8 (3)
H111—O11—H211	111 (3)	C2—C1—H1	119.00
Cr1—O11—H111	109.4 (18)	C5—C1—H1	119.00
H112—O12—H212	110 (3)	C1—C2—H2	119.00
Cr1—O12—H212	115 (2)	C3—C2—H2	119.00
Cr1—O12—H112	112.8 (16)	C2—C3—H3	122.00
Cr2—O23—C24	113.81 (15)	N12—C3—H3	122.00
Cr2—O24—C23	113.37 (14)	C5—C4—H4	120.00
Cr2—O25—C21	114.67 (15)	N12—C4—H4	120.00
Cr2—O26—C22	112.90 (16)	N22—C6—C10	119.9 (3)
H121—O21—H221	113 (3)	N22—C7—C8	117.5 (3)
Cr2—O21—H221	115.6 (16)	C7—C8—C9	120.2 (3)
Cr2—O21—H121	111 (2)	C8—C9—C10	121.7 (3)
H122—O22—H222	109 (2)	N21—C10—C9	123.9 (3)
Cr2—O22—H122	113 (2)	C6—C10—C9	115.9 (3)
Cr2—O22—H222	112.0 (18)	N21—C10—C6	120.2 (3)
C3—N12—C4	124.8 (3)	N22—C6—H6	120.00
H11A—N11—H11B	120.00	C10—C6—H6	120.00
C5—N11—H11A	120.00	N22—C7—H7	121.00
C5—N11—H11B	120.00	C8—C7—H7	121.00
C4—N12—H12	118.00	C7—C8—H8	120.00
C3—N12—H12	118.00	C9—C8—H8	120.00
C6—N22—C7	124.7 (3)	C8—C9—H9	119.00
C10—N21—H21B	120.00	C10—C9—H9	119.00
			
O11—Cr1—O13—C12	−91.40 (16)	Cr2—O23—C24—C23	−1.1 (2)
O12—Cr1—O13—C12	89.16 (16)	Cr2—O23—C24—O30	178.4 (2)
O14—Cr1—O13—C12	−1.46 (16)	Cr2—O24—C23—O28	175.3 (2)
O16—Cr1—O13—C12	179.52 (16)	Cr2—O24—C23—C24	−4.8 (2)
O11—Cr1—O14—C11	92.22 (16)	Cr2—O25—C21—C22	1.2 (2)
O12—Cr1—O14—C11	−88.28 (16)	Cr2—O25—C21—O29	179.8 (2)
O13—Cr1—O14—C11	1.34 (16)	Cr2—O26—C22—C21	2.5 (3)
O15—Cr1—O14—C11	−176.66 (15)	Cr2—O26—C22—O27	−177.3 (2)
O11—Cr1—O15—C13	−91.84 (16)	C3—N12—C4—C5	0.1 (5)
O12—Cr1—O15—C13	87.64 (16)	C4—N12—C3—C2	−1.2 (5)
O14—Cr1—O15—C13	177.91 (15)	C7—N22—C6—C10	0.1 (5)
O16—Cr1—O15—C13	−2.94 (16)	C6—N22—C7—C8	−0.3 (5)
O11—Cr1—O16—C14	91.27 (16)	O17—C11—C12—O19	−0.5 (4)
O12—Cr1—O16—C14	−88.20 (16)	O14—C11—C12—O13	−0.2 (3)
O13—Cr1—O16—C14	−177.90 (16)	O17—C11—C12—O13	179.1 (2)
O15—Cr1—O16—C14	0.05 (16)	O14—C11—C12—O19	−179.7 (2)
O25—Cr2—O26—C22	−1.51 (16)	O15—C13—C14—O18	175.1 (2)
O21—Cr2—O23—C24	85.88 (16)	O20—C13—C14—O18	−4.9 (4)
O22—Cr2—O23—C24	−92.75 (16)	O15—C13—C14—O16	−4.9 (3)
O24—Cr2—O23—C24	−1.11 (15)	O20—C13—C14—O16	175.1 (2)
O25—Cr2—O23—C24	175.60 (16)	O25—C21—C22—O26	−2.5 (3)
O21—Cr2—O24—C23	−88.89 (16)	O29—C21—C22—O26	178.8 (2)
O22—Cr2—O24—C23	90.59 (16)	O29—C21—C22—O27	−1.4 (4)
O23—Cr2—O24—C23	3.45 (15)	O25—C21—C22—O27	177.2 (2)
O26—Cr2—O24—C23	−178.25 (15)	O28—C23—C24—O30	4.4 (4)
O21—Cr2—O25—C21	−89.89 (16)	O28—C23—C24—O23	−176.1 (2)
O22—Cr2—O25—C21	90.74 (16)	O24—C23—C24—O30	−175.5 (2)
O23—Cr2—O25—C21	178.12 (16)	O24—C23—C24—O23	4.0 (3)
O26—Cr2—O25—C21	0.05 (17)	C5—C1—C2—C3	−1.5 (5)
O21—Cr2—O26—C22	88.14 (17)	C2—C1—C5—C4	0.3 (5)
O22—Cr2—O26—C22	−93.17 (17)	C2—C1—C5—N11	−179.2 (3)
O24—Cr2—O26—C22	175.31 (16)	C1—C2—C3—N12	1.9 (5)
Cr1—O13—C12—C11	1.3 (2)	N12—C4—C5—N11	179.9 (3)
Cr1—O13—C12—O19	−179.2 (2)	N12—C4—C5—C1	0.4 (4)
Cr1—O14—C11—C12	−1.0 (2)	N22—C6—C10—N21	180.0 (3)
Cr1—O14—C11—O17	179.8 (2)	N22—C6—C10—C9	0.5 (4)
Cr1—O15—C13—O20	−175.2 (2)	N22—C7—C8—C9	−0.1 (5)
Cr1—O15—C13—C14	4.8 (2)	C7—C8—C9—C10	0.8 (5)
Cr1—O16—C14—C13	2.4 (2)	C8—C9—C10—N21	179.6 (3)
Cr1—O16—C14—O18	−177.6 (2)	C8—C9—C10—C6	−1.0 (5)

Symmetry codes: (i) −*x*+1, *y*+1/2, −*z*+1/2; (ii) −*x*+1, −*y*+1, −*z*+1; (iii) *x*+1, *y*, *z*; (iv) −*x*+2, −*y*+1, −*z*+1; (v) −*x*+1, *y*−1/2, −*z*+1/2; (vi) *x*+1, −*y*+1/2, *z*+1/2; (vii) −*x*+1, −*y*, −*z*+1; (viii) *x*−1, *y*, *z*; (ix) *x*, −*y*+1/2, *z*+1/2; (x) *x*, *y*−1, *z*; (xi) −*x*, *y*−1/2, −*z*+1/2; (xii) *x*, −*y*+1/2, *z*−1/2; (xiii) *x*, *y*+1, *z*; (xiv) −*x*, *y*+1/2, −*z*+1/2; (xv) *x*−1, −*y*+1/2, *z*−1/2.

### Hydrogen-bond geometry (Å, º)

**Table:**

*D*—H···*A*	*D*—H	H···*A*	*D*···*A*	*D*—H···*A*
N11—H11*A*···O25^xii^	0.86	2.22	3.047 (4)	161
N11—H11*B*···O22^i^	0.86	2.46	3.139 (4)	136
N12—H12···O18^v^	0.86	2.30	2.998 (3)	139
N12—H12···O20^v^	0.86	2.16	2.896 (3)	144
N21—H21*A*···O13^ii^	0.86	2.31	3.142 (3)	163
N21—H21*B*···O11^viii^	0.86	2.42	3.201 (4)	151
N22—H22···O28^xiii^	0.86	2.38	3.121 (3)	145
N22—H22···O30^xiii^	0.86	2.05	2.755 (3)	138
O11—H111···O27^iii^	0.84 (3)	2.30 (3)	3.103 (3)	161 (2)
O11—H111···O29^iii^	0.84 (3)	2.19 (2)	2.749 (3)	123 (2)
O11—H211···O18^iv^	0.84 (1)	1.95 (2)	2.777 (3)	173 (3)
O12—H112···O27	0.84 (2)	1.85 (2)	2.684 (3)	176 (2)
O12—H212···O28^i^	0.84 (1)	1.78 (1)	2.619 (2)	172 (3)
O21—H121···O20^v^	0.84 (2)	1.85 (1)	2.682 (3)	176 (3)
O21—H221···O17^viii^	0.84 (2)	1.85 (2)	2.681 (3)	177 (2)
O22—H122···O23^vii^	0.85 (2)	1.94 (2)	2.780 (2)	173 (3)
O22—H222···O19	0.84 (2)	1.85 (2)	2.646 (3)	157 (3)

Symmetry codes: (i) −*x*+1, *y*+1/2, −*z*+1/2; (ii) −*x*+1, −*y*+1, −*z*+1; (iii) *x*+1, *y*, *z*; (iv) −*x*+2, −*y*+1, −*z*+1; (v) −*x*+1, *y*−1/2, −*z*+1/2; (vii) −*x*+1, −*y*, −*z*+1; (viii) *x*−1, *y*, *z*; (xii) *x*, −*y*+1/2, *z*−1/2; (xiii) *x*, *y*+1, *z*.
